# Automaticity of social cues: The influence of limiting cognitive resources on head orientation cueing

**DOI:** 10.1038/s41598-018-28548-x

**Published:** 2018-07-06

**Authors:** Troy A. W. Visser, Ashton Roberts

**Affiliations:** School of Psychological Science, The University of Western Australia, Crawley, WA Australia

## Abstract

Our ability to communicate effectively often relies on being able to shift our focus of attention to align with that of another person. This so-called “social attention” reflects the use of cues such as gaze, pointing and head orientation to infer the attentional focus of others. An important, but unresolved, question is whether these socially relevant cues automatically direct attention in observers, or whether cognitive resources shape this process. An additional issue is that existing work has almost exclusively examined eye gaze cues, thus potentially limiting the generalizability of this work across types of social cues. To examine these issues, the present research investigates the influence of limiting resource availability (using a concurrent memory load) on the ability of an oriented head cue to direct attention. The results indicate that reducing resource availability increases the impact of the head cue on attentional orienting – the opposite pattern to that obtained with gaze cues. This outcome suggests that resource availability does not affect all social cues the same, and that caution is warranted in drawing broad conclusions about mechanisms underlying social cueing of attention without appropriate comparisons across multiple types of social cues.

## Introduction

Social interactions at the coffee shop, in the boardroom, and around the home all depend upon our ability to rapidly understand the focus of attention for each speaker and to shift our own attention accordingly. Communication of attentional focus from one person to another – a process referred to as “social attention”^1,2^ – arises from a complex web of language and body actions including gaze, head movements, and body posture.

One of the first studies to examine how cues to social attention influence orienting was conducted by Friesen and Kingstone^3^; see also^4,5^. These researchers presented participants with schematic images of faces with the eyes looking left, right, or straight ahead. Faces with averted gaze aligned with one of two possible locations for upcoming targets, but did not predict target location. Nevertheless, although aware of the non-predictive nature of gaze, observers still responded faster and more accurately to targets in the direction of gaze than when gaze was straight ahead or oriented away from the target.

In the ensuing twenty years since this seminal finding (see^6^ for a review), significant effort has been devoted to understanding whether social cues, such as gaze, direct attention exogenously (i.e., involuntarily, automatically) or endogenously (i.e., voluntarily, guided by effortful cognitive control processes). One focus of this work has been to compare characteristics of cueing arising from eye gaze to that arising from cues thought to be chiefly exogenous (e.g., visual onsets presented at potential target locations) or chiefly endogenous (e.g., central arrows pointing to potential target locations). For example, Friesen, Ristic and Kingstone^7^ asked participants to respond to targets presented above, below, left or right of a central face that gazed either right or left prior to target onset. Participants were informed that the target would appear in the location opposite to the direction of gaze on 75% of trials, in the direction of gaze on 8% of trials, and at the remaining locations on 17% of trials. In this way, gaze direction was made counter-predictive. Performance was then compared with identical trials in which a central arrow pointed left or right. The critical result was that gaze cues benefited performance for targets in the direction of gaze at short inter-target intervals (~100 ms), whereas such benefits were never seen for arrow cues. This was taken as evidence that gaze cues trigger exogenous, rather than endogenous, attentional shifts.

On the other hand, several pieces of evidence suggest that gaze cues may be linked to endogenous processes as well. Like Friesen *et al*.^7^, Tipples^8^ compared the effect of counterproductive gaze, arrow, and abrupt-onset cues on target detection. Unlike Friesen *et al*.^7^, however, Tipples found similar patterns of target responses for both gaze and arrow cues. Moreover, while self-reported attentional control abilities were positively correlated with performance for gaze and arrow cues, this relationship was absent for abrupt-onset cues. Vecera and Rizzo^9^ examined gaze and onset cueing in a patient with orbitofrontal lesions who suffered broad-based difficulties in goal-directed behaviour. While this patient showed intact gaze cueing, gaze failed to produce an orienting response. In light of their results, both Tipples^8^ and Vecera and Rizzo^9^ concluded that gaze cues were significantly impacted by endogenous attentional control processes, rather than being exogenous.

Such conflicting results point to a potential problem when attempting to establish mechanisms of social cueing by comparing performance across cue types. Namely, arrow and abrupt-onset cues may activate at least some overlapping mechanisms rather than being exclusively endogenous or exogenous in nature. For example, it is well known that the ability of onsets to direct attention is modulated by whether they share task-relevant properties^10,11^. and both arrow and onset cues activate overlapping dorsal areas in the brain^12^.

With this in mind, an arguably more fruitful approach to understanding the mechanisms underlying social cueing has been to draw on the notion of automaticity to test the extent to which these cues can influence performance. The concept of automaticity in cognitive psychology has a long and substantial history, with extensive debates as to how one can determine whether a process is automatic or volitional (e.g.^13–19^. Common amongst these taxonomies is that an automatic process should: (a) occur rapidly, (b) be mandatory, and (c) be unaffected by concurrent cognitive demands (see^20^ for a discussion of these criteria with regards to face perception).

Considering these criteria in turn, there is clearly ample evidence that gaze cueing occurs rapidly, as numerous studies have shown that benefits for targets in the direction of gaze arise around 100 ms from the onset of the cue (e.g.^3,5,7,8,21,22^. There is also clear evidence that gaze cues mandatorily orient attention of observers. Initial studies showed that cueing effects arose despite the fact that observers knew that the cues did not predict target location^3,5^. Moreover, as reviewed above, even when gaze direction validly predicts that a subsequent target will appear at another location, targets in the direction of gaze are still advantaged^7,8,22^.

However, investigations of whether gaze cues are unaffected by cognitive demands have yielded more disparate results. Initial studies by Hayward and Ristic^23^ and Law, Langton and Logie^24^ suggested that concurrent memory load did not influence the magnitude of cueing. Law *et al*.^24^ manipulated cognitive demands by asking participants to remember one (low demand) or five (high demand) letters prior to the presentation of a conventional gaze cueing trial. They found that target responses were slowed in the high demand condition, but this did not influence the magnitude of gaze cueing. More recently, however, Bobak and Langton^25^ found significantly less gaze cueing when the cognitive demands of the concurrent task were higher (generating random numbers) than when the demands were lower (reciting the numbers 1–9 in order). This suggests that gaze cueing is not immune to interference from concurrent cognitive demands, implying that social cues may only be partially automatic as measured against this conventional criterion.

Before drawing such a conclusion about social cues more generally, however, it is critical to ascertain the effects of cognitive demands on social cues other than gaze. For example, pointing gestures are known to effectively guide the attention of observers to environmental stimuli^26,27^. Similarly, body posture is also involved in the computation of social attention. Cells in the STS of macaque monkeys that are sensitive to downward gaze also show large responses to human faces looking downwards and larger responses to bodies in a quadrupedal position (consistent with attending downwards) than in an upright bipedal position (consistent with attending forwards^1^. Finally, Langton and Bruce^5,27,28^ found human observers responded more quickly and accurately to targets aligned with the direction of an oriented head than targets presented in another location. This was true both when the interval between the head and target were brief and when participants knew head orientation did not predict target location.

In the present work, we probe the extent to which head orientation yields automatic shifts of attention by examining the effect of concurrent cognitive demands on the magnitude of head orientation cueing. This is a particularly important question for two reasons. First, it directly addresses the issue of whether a social cue other than gaze – namely, head orientation – is processed automatically. Second, it will yield important information about the relative similarities between the different types of social cues in directing attention. A high degree of similarity between the pattern of results obtained here and patterns from earlier studies using eye gaze would suggest orienting of attention arising from different social cues might be subsumed by broadly similar mechanisms. Conversely, differences in the patterns of effects arising from eye gaze cues in past studies and head orientation cues might suggest that social cueing is the product of a heterogeneous set of mechanisms that vary with the type of social cue being attended.

To explore these issues, in Experiment 1, we investigated whether attentional shifts in response to head orientation are sensitive to concurrent working memory load^23,24^ by requiring participants to memorize either 2 or 6 letters prior to the onset of a target discrimination task, and then report whether a single probe letter was part of the to-be-remembered set. In accordance with the criteria for automaticity outlined above, an effect of memory load on cueing would imply that underlying processes were impacted by concurrent cognitive demands.

## Experiment 1

### Method

#### Participants

Twenty-five participants (22 females, mean age: 22.8 years) were recruited by word-of-mouth and from introductory psychology classes at the University of Western Australia and received credit towards course completion where applicable. All participants had normal or corrected-to-normal vision, and were naïve to the purpose of the experiment.

#### Ethical approval and informed consent

This study was approved under the University of Western Australia Human Research Ethics Board (RA/4/1/5247). The experiment was conducted in accordance with the relevant guidelines and regulations, and all participants signed informed consent documents upon commencing the protocol.

#### Data availability statement

The datasets generated during and/or analysed during this experiment are available from the corresponding author on reasonable request.

#### Apparatus and materials

**Figure 1:**
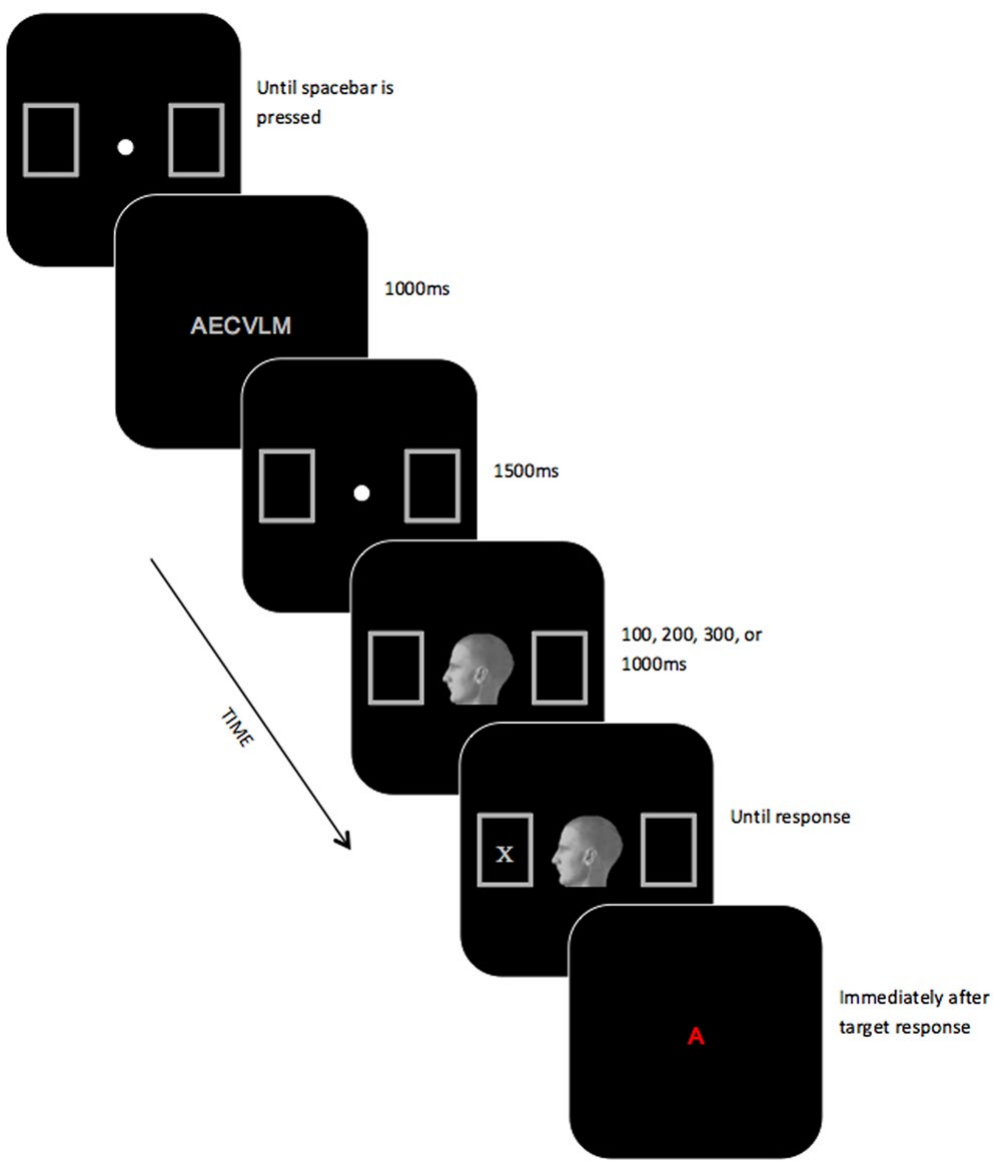
Schematic diagram of an event sequence on a typical trial with a six-item memory load in Experiment 1. Stimuli are not to scale. The fixation dot, head cue, and target are disproportionately enlarged to show detail.

Presentation software (Neurobehavioural Systems, 2014) was used to present stimuli and collect responses. Participants were seated ~60 cm from a 24′′ LCD monitor (100 Hz refresh). As can be seen in Fig. 1, cue stimuli were digitised, greyscale images of a head (~4° square) that was facing left or right. The target consisted of a dark grey (RGB: 136, 136, 136) “X” or “+” symbol (~1° square) that appeared inside one of two dark gray outlined placeholder boxes (~2° square) positioned 10° to the left or right of a yellow fixation dot (RGB: 255, 255, 0) located at the centre of the display. Either two or six light gray, upper-case letters (RGB: 167, 167, 167; approximately 1° square) were used for the memory load task. These letters were presented in 28 pt Arial font and were randomly chosen from all possible letters in the English alphabet except for I, O, P, Q, X, and Z, with the constraint that the same letter could not be used more than once on a trial.

#### Procedure

A schematic outline of the sequence of events on a typical trial is shown in Fig. 1. Trials began with a fixation dot presented in the center of the display accompanied by two empty placeholder boxes. This fixation dot remained visible until participants had pressed the space bar to start the trial. Next, the memory set consisting of two or six different randomly-chosen letters was presented at the center of the display for 1000 ms. This was followed by a second presentation of the fixation dot and accompanying placeholder boxes for 1500 ms. The fixation dot was then replaced by an oriented head, which was followed immediately by a target presented inside one of the placeholders. The oriented head was displayed for one of four durations to yield a cue-target onset asynchrony (CTOA) of 100, 200, 300, or 1000 ms. These CTOAs were chosen to overlap with those used in previous gaze cueing studies^3,5,7–9^. As soon as participants had detected the appearance of the target, they were instructed to identify it as quickly and accurately as possible by pressing a marked key. As soon as the target response was made, a single red probe letter was presented in the center of the display. On half of trials, this probe was identical to a letter from the memory set, while on the other half, the probe was not part of the memory set. Participants were instructed to press the left arrow key if the letter was part of the memory set, or the right arrow key if it was not, and to prioritize accuracy over speed. Following this probe response, the next trial began with the presentation of the central fixation dot and accompanying placeholder boxes.

The experiment consisted of 576 trials divided into 9 blocks of 64 trials each. Within each block, every possible combination of head orientation (left or right), CTOA (100, 200, 300, 1000 ms), target identity (X or+), target location (left or right) and memory load (two or six letters) was presented. This yielded a total of 36 validly-cued trials (in which the head was oriented in the direction of the target location) and 36 invalidly-cued trials (in which the head was oriented in the opposite direction of the target location) per combination of CTOA and memory load across the duration of the experiment. Participants were instructed to take a rest break between each block of trials and during the initial fixation display on each trial when needed in order to maintain vigilance.

## Results

Mean accuracy on the memory load task was calculated separately as a function of memory load and CTOA. Mean target identification accuracy and response time (RT) were calculated separately as a function of memory load, CTOA and cue validity only on trials in which the memory load task was performed correctly. Mean target RTs were calculated separately as a function of memory load, CTOA and cue validity only on trials in which the memory load and target identification tasks were performed correctly. Data from trials with RTs less than 200 ms or greater than 2000ms (0.08%) were omitted from further analysis.

Mean accuracy on the memory load task was analysed using a Load (2 vs. 6 items) x CTOA (100, 200, 300, 1000 ms) repeated-measures analysis of variance (ANOVA). This revealed only a significant main effect of Load, *F*(1, 24) = 23.40, *p* < 0.001, η_p_^2^ = 0.46, indicating that performance was greater when two items had to be remembered (91.7%) than when six items had to be remembered (83.9%). This confirms that cognitive requirements increased with memory load. No other main effects or interactions were significant (*p* > 0.20, η_p_^2^ < 0.06).

Mean target identification accuracy was analysed using a Load x CTOA x Validity (valid vs. invalid cues) ANOVA which revealed a CTOA x Validity interaction, *F*(3, 66) = 4.04, *p* < 0.02, η_p_^2^ = 0.16. However, closer examination of the data underlying this interaction suggested no interpretable pattern, likely because identification accuracy was near ceiling level (mean: 97.8%; range: 97.0–99.0). No other main effects or interactions were significant (*p* > 0.39, η_p_^2^ < 0.04).

**Figure 2:**
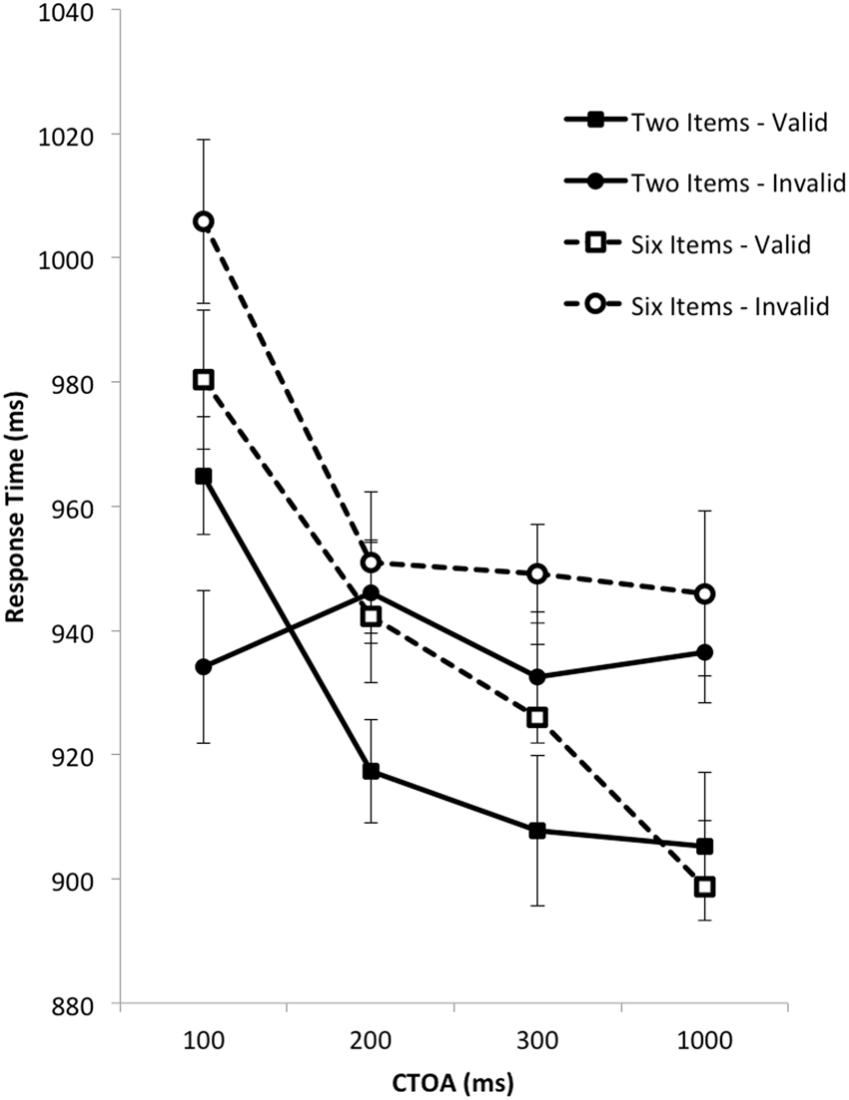
Mean response times in Experiment 1 plotted as a function of CTOA. Solid lines represent performance in the condition in which two items had to be remembered. Dashed lines represent performance in the condition in which six items had to be remembered. Square symbols represent performance on valid trials. Circle symbols represent performance on invalid trials. The error bars represents 95% within-subjects confidence interval computed as per O’Brien and Cousineau^31^.

Mean target identification RTs were analysed using an identical Load x CTOA x Validity ANOVA which revealed significant main effects of Load, *F*(1, 24) = 5.78, *p* = 0.024, η_p_^2^ = 0.19, CTOA, *F*(3, 72) = 15.71, *p* < 0.001, η_p_^2^ = 0.39, and Validity, *F*(1, 24) = 13.74, *p* = 0.001, η_p_^2^ = 0.36. As can be seen in Fig. 2, overall RTs were faster when fewer items needed to be

remembered, declined with increasing CTOA, and were faster on validly-cued trials than on invalidly-cued trials. There was also a significant CTOA x Validity interaction, *F*(3, 72) = 3.17, *p* = 0.029, η_p_^2^ = 0.12, and most importantly a significant Load x CTOA x Validity interaction, *F*(3, 72) = 3.02, *p* = 0.035, η_p_^2^ = 0.11, suggesting that memory load mediated the impact of head orientation cues on performance.

To explore this possibility further, we conducted CTOA x Validity ANOVAs separately for each Load. When memory load was high, this analysis revealed only a significant main effect of CTOA, *F*(3, 72) = 12.25, *p* < 0.001, η_p_^2^ = 0.34, and a significant main effect of Validity, *F*(1, 24) = 13.24, *p* = 0.001, η_p_^2^ = 0.36. These findings confirm the graphical evidence from Fig. 2 showing a decline in RTs across CTOA, coupled with reliable benefits for validly-cued targets emerging at the 100 ms CTOA. In contrast, when memory load was low, the same analysis showed a main effect of CTOA, *F*(3, 72) = 3.75, *p* = 0.015, η_p_^2^ = 0.14, and a significant CTOA x Validity interaction, *F*(3, 72) = 5.74, *p* = 0.001, η_p_^2^ = 0.19. These findings confirm the graphical evidence from Fig. 2 showing an overall decline in RTs across CTOA, coupled with a deficit in performance for validly-cued targets at the 100 ms CTOA [*t*(24) = 2.35, *p* = 0.028], followed by benefits for validly-cued targets at all other CTOAs.

On the basis of this result, it seems that attention shifts induced by head orientation are influenced by concurrent cognitive demands at brief CTOAs, and thus fail to meet this criterion for automaticity. Moreover, our results using head orientation cues differ from earlier studies using gaze cues. First, we found that lower memory load interfered with the benefits of valid head orientation cues, whereas previous studies found higher memory load interfered with benefits of valid gaze cues^25^. Second, unlike earlier work by Langton and Bruce^5^, cueing benefits arising from valid head orientation cues persisted at CTOAs of 1000 ms, rather than dissipating quickly. Interestingly, this finding mirrors results using eye gaze cues where some studies show prolonged benefits^8,21^ while others do not^22,25^. These findings suggest both similarities and differences between gaze and orientation cues that argue against the notion that gaze cues can be considered broadly representative of all social cues.

To confirm these conclusions, however, it is desirable to obtain converging evidence for the effects seen in Fig. 1 using a different paradigm to vary cognitive demands. In this regard, it is notable that cognitive demands modulated gaze cueing when participants had to perform a demanding random number generation task^25^ but not when they had to memorize a set of items^24^ as in Experiment 1. This opens up the possibility that varying the type and difficulty of cognitive demands, as in these earlier studies, might also differentially influence the nature of head orientation cueing.

To test this possibility, in Experiment 2, we asked participants to complete a demanding calculation task prior to the appearance of the head orientation cue and subsequent target. We modelled our task after that used by Dux, Visser, Goodhew and Lipp^29^. At the start of each trial, participants were presented with four sequential digits that they used to complete a sequence of addition and subtraction operations. The last digit was then followed after at a short or long interval by the oriented head and target stimuli used in Experiment 1. It was expected that the cognitive demands of the calculation task would be greater at the shorter interval than at the longer interval, providing an opportunity to assess the impact of varying cognitive demands on the magnitude of cueing. Based on the results of Experiment 1, it would be expected that the longer interval (with relatively lower cognitive demands) should yield evidence for reduced cueing benefits.

## Experiment 2

### Method

#### Participants

Twenty-eight participants (20 females, median age: 21.0 years) were recruited by word-of-mouth and from introductory psychology classes at the University of Western Australia and received credit towards course completion where applicable. All participants had normal or corrected-to-normal vision, were naïve about the purpose of the experiment, and had not participated in the previous experiment.

#### Ethical approval and informed consent

This study was approved under the University of Western Australia Human Research Ethics Board (RA/4/1/5247). The experiment was conducted in accordance with the relevant guidelines and regulations, and all participants signed informed consent documents upon commencing the protocol.

#### Data availability statement

The datasets generated during and/or analysed during this experiment are available from the corresponding author on reasonable request.

#### Apparatus and materials

The apparatus and materials were identical to Experiment 1, except for the removal of letters used in the memory load task. Four light grey, upper-case digits (RGB: 167, 167, 167; approximately 1^o^ square) were used for the calculation task. These digits were presented in 28 pt Arial font and were randomly chosen from 1–9, with the constraint that the same digit could not be used consecutively during a trial.

#### Procedure

**Figure 3:**
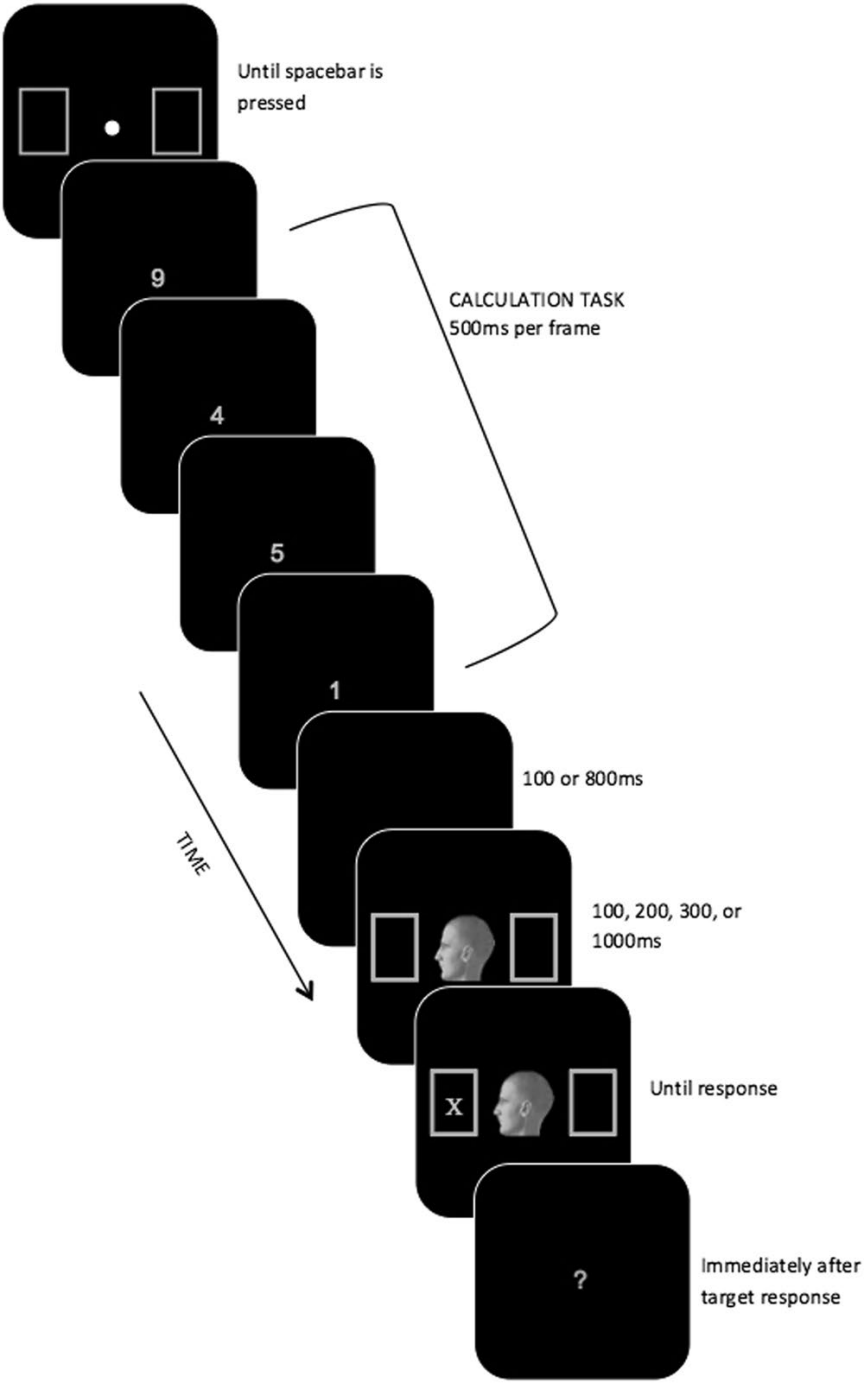
Schematic diagram of an event sequence on a typical trial for Experiment 2. Stimuli are not to scale. The fixation dot, head cue, and target are disproportionately enlarged to show detail.

A schematic outline of the sequence of events on a typical trial is shown in Fig. 3. Trials began with a fixation dot presented in the center of the display accompanied by two empty placeholder boxes. This fixation dot remained visible until participants had pressed the space bar to start the trial at which point the fixation disappeared while the placeholder boxes remained on the display. Next, a sequence of four digits was presented one-at-a-time at fixation. Each digit was presented for 500 ms, and separated from the next digit by a 500 ms inter-stimulus interval. As each digit appeared, participants were instructed to complete an arithmetic computation (digit 1+ digit 2 - digit 3+ digit 4). Following the final digit, there was another pause for either 100 (short interval) or 800 (long interval) ms. Then an oriented head and target were presented exactly as in Experiment 1. As soon as participants had detected the appearance of the target, they were instructed to identify it as quickly and accurately as possible by pressing a marked key. They were then prompted to report whether the calculation yielded an odd or even number, by pressing a different marked key. They were instructed to make this decision as accurately as possible without speed pressure. After this response was made, the next trial began with the presentation of the central fixation dot and accompanying placeholder boxes.

The experiment consisted of 576 trials divided into 9 blocks of 64 trials each. Within each block, every possible combination of head orientation (left or right), CTOA (100, 200, 300, 1000 ms), target identity (X or +), target location (left or right) and interval between the final digit and oriented head cue (henceforth referred to as interval: 100 or 800 ms). This yielded a total of 36 validly-cued trials (in which the head was oriented in the direction of the target location) and 36 invalidly-cued trials (in which the head was oriented in the opposite direction of the target location) per combination of CTOA and interval across the duration of the experiment. Participants were instructed to take a rest break between each block of trials and during the initial fixation display on each trial when needed in order to maintain vigilance.

## Results

Mean accuracy on the computation task was calculated separately as a function of interval and CTOA. Mean target identification accuracy and RT were calculated separately as a function of interval, CTOA and cue validity only on trials in which the computation task was performed correctly. Mean target RTs were calculated separately as a function of interval, CTOA and cue validity only on trials in which the computation and target identification tasks were performed correctly. On the basis of these calculations, data from one participant who scored more than 2.5 standard deviations below the mean on the computation task was omitted from further analysis. Data from trials with RTs less than 200 ms or greater than 2000ms (0.03%) were also omitted from further analysis.

Mean accuracy on the computation task was analysed using an Interval (100 vs. 800 ms) × CTOA (100, 200, 300, 1000 ms) repeated-measures ANOVA. This revealed only a significant main effect of Interval, *F*(1, 26) = 4.38, *p* = 0.046, η_p_^2^ = 0.14, indicating that performance was greater when the interval between the computation task and cue was longer (88.6%) than when it was shorter (86.8%). This suggests that performance on the computation task decreased slightly when it was followed immediately by the head cue, implying some interference occurred between the tasks. No other main effects or interactions were significant (*p* > 0.05, η_p_^2^ < 0.10).

Mean target identification accuracy was analysed using an Interval x CTOA x Validity (valid vs. invalid cues) ANOVA which revealed no significant main effects or interactions (*p* > 0.05, η_p_^2^ < 0.10). This likely reflects the fact that target identification accuracy was near ceiling level (95.8%; range 94.3–97.4%).

**Figure 4:**
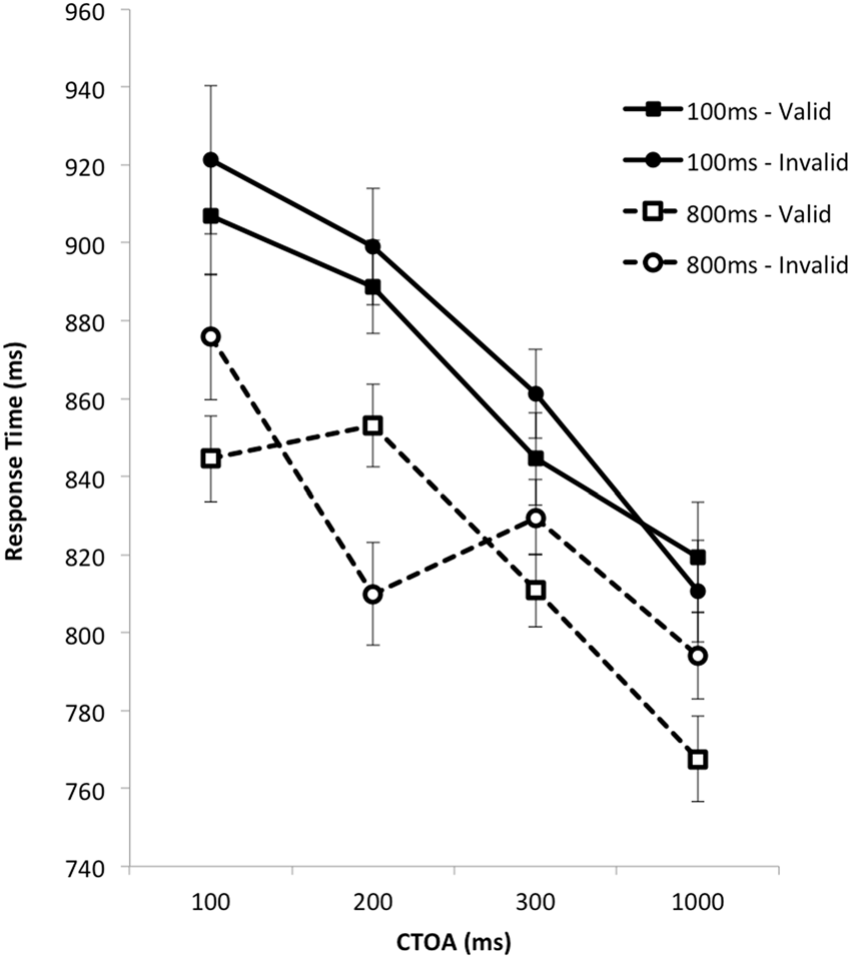
Mean response times in Experiment 2 plotted as a function of CTOA. Solid line represented performance in the condition in which the interval between the final number and the head cue was short (100 ms). Dashed line represents performance in the condition in which the interval was long (800 ms). Square symbols represent the performance on valid trials. Circle symbols represent performance on invalid trials. The error bars represent 95% within-subjects confidence intervals computed as per O’Brien and Cousineau^31^.

Mean target identification RTs were analysed using an identical Interval x CTOA x Validity ANOVA which revealed significant main effects of Interval, *F*(1, 26) = 34.36, *p* < 0.001, η_p_^2^ = 0.57, CTOA, *F*(3, 78) = 32.87, *p* < 0.001, η_p_^2^ = 0.56, and Validity, *F*(1, 26) = 5.69, *p* = 0.025, η_p_^2^ = 0.18. As can be seen in Fig. 4, overall RTs were faster when the interval between the computation task and the cue was longer (i.e. when cognitive demand was lower), declined with increasing CTOA, and were faster on validly-cued trials than on invalidly-cued trials.

Critically, there was also a significant Interval x CTOA x Validity interaction, *F*(3, 72) = 3.02, *p* = 0.035, η_p_^2^ = 0.11, suggesting that the interval between the demanding computation task and the cue differentially affected the impact of the oriented head on target RTs across CTOAs. No other main effects or interactions were significant (*p* > 0.14, η_p_^2^ < 0.07).

To explore the three-way interaction further, we conducted CTOA x Validity ANOVAs separately at each Interval. When the interval between the calculation task and head orientation cue was short, this analysis revealed a significant main effects of CTOA, *F*(3, 78) = 17.24, *p* < 0.001, η_p_^2^ = 0.40. This finding confirms the graphical evidence from Fig. 4 showing a decline in RTs across CTOA, coupled with non-significant benefits for validly-cued targets. In contrast, when the interval between the calculation task and head orientation was long, the same analysis showed a main effect of CTOA, *F*(3, 78) = 12.31, *p* < 0.001, η_p_^2^ = 0.32, and a significant CTOA x Validity interaction, *F*(3, 78) = 6.12, *p* = 0.001, η_p_^2^ = 0.19. These findings confirm the graphical evidence from Fig. 4 showing an overall decline in RTs across CTOA, coupled with a deficit in performance for validly-cued targets at the 200 ms CTOA [*t*(28) = 2.75, *p* = 0.011], with benefits for validly-cued targets at all other CTOAs.

The principal findings of Experiment 2 replicate those of Experiment 1. At brief CTOAs, reducing cognitive demand reversed cueing benefits for validly-cued targets. That said, this effect appeared about 100 ms later in Experiment 2 than in Experiment 1. This later onset may reflect the fact that the calculation task relied more heavily on high-level cognitive operations and less on storage capacity than the recognition task used in Experiment 1. Additionally, as in Experiment 1, cueing benefits also persisted until the 1000 ms CTOA, at least at the longer interval, *t*(28) = 2.17, *p* = 0.004. This provides some additional evidence that cueing arising from head orientation may reliably persist longer than that arising from gaze cues, at least under the experimental conditions employed here.

## General Discussion

Human interactions are greatly aided by our ability to process social attention cues, such as gaze direction, head orientation, and pointing, in order to establish and match the attentional focus of another individual. A persistent question in the literature is whether humans orient attention in response to such cues automatically, or whether this process is shaped by other cognitive demands. To date, research on this question, using only eye gaze cues, has implied that social cues are at least partly automatic, leading to rapid shifts of attention that are resistant to task instructions or parameters that discourage such shifts^3,8,21–23^. However, it is still unclear whether processing of social cues other than gaze is unaffected by concurrent task demands^24,25^ and whether results obtained using gaze cues are similar to other found using other social cues such as head orientation. These two questions were the focus of the present work.

Across two experiments, we found that head orientation yielded relatively rapid attentional orienting that was moderated by concurrent cognitive demands. However, the pattern of these effects differed from that seen in past studies. Whereas valid eye gaze cues were less effective at orienting attention when presented concurrently with a more demanding task^25^, valid head orientation cues were less effective at orienting attention when presented concurrently with a less demanding task. This disjunction between the influence of cognitive demands on orienting to the two types of cues strongly suggests that social orienting does not arise from a singular set of processes but rather multiple processes that at least partially vary depending on the type of social cue.

An additional finding was that benefits for validly-cued targets persisted for 1000 ms after cue presentation despite the fact that participants knew that head orientation was non-predictive. Such persistent advantages are not typically found when uninformative gaze cues are presented alone^3,22,25^, although they have been obtained under more complex conditions with counterproductive gaze cues^8^ or when gaze cues follow an initial cue that perfectly predicts target location^21^. This contrast between orientation and gaze cues provides further suggestive evidence that the same processes do not mediate the two social cues. It also implies that at least some social cues do not just rapidly influence attentional focus, but may also periodically lead to volitional shifts of attentional focus despite knowledge that social cues are unhelpful for the task at hand. While this would not be a unique property of social cues – similar effects were reported by Galfano *et al*.^21^ with arrow cues – the origins of such persistent attentional shifts nonetheless deserve further investigation. One possibility is that prolonged cueing benefits may reflect response preparedness^30^.

In both experiments, increased cognitive demands led to significantly slower responses to validly-cued targets than to invalidly-cued targets at brief CTOAs. Closer examination of Figs 2 and 4 suggests that when cognitive demands were greater (i.e. at shorter CTOAs), validly-cued targets were responded to slower than those presented at longer CTOAs. In comparison, invalidly cued targets were responded to at much the same speed across these CTOAs. For example, examination of the low memory load condition in Fig. 2 clearly shows that RTs to invalidly-cued targets are similar across all CTOAs, whereas RTs to validly-cued targets are similar across the three longer RTs and much slower at the earliest CTOA. Similarly, examination of long-interval condition in Fig. 4 shows RTs to invalidly cued targets remaining relatively constant across the 200–1000 ms CTOAs, whereas RTs to validly cued targets declined steeply over this period. In sum, then, it would appear that increasing cognitive demands might have slowed attentional shifts to validly-cued targets rather than speeding attentional shifts to invalidly-cued targets. Of course, this explanation is ad-hoc and needs to be carefully evaluated in future replications.

Before concluding, it is also important to point out some possible limitations of the present work. Our results provide an unambiguous answer to our first research question: concurrent memory load modulates head orientation cueing. However, the validity of subsequent comparisons between our findings and those of prior gaze-cueing studies could be limited because they are based on experimental findings from different participants and methodologies. Logically, therefore, diverging experimental results could stem from these differences rather than the nature of the social cue. We think this option is unlikely because we chose CTOAs, concurrent tasks, and stimuli that were representative of, or identical to, those used in earlier gaze cue studies. We are currently conducting experiments to confirm dissociations between types of social cues by comparing performance from the same participants across social attention tasks that differ only in cue type.

In conclusion, the present experiments investigated a long-standing issue concerning the ability of social cues to automatically guide attention. Using head orientation as a source of social attention information, the present results affirmed earlier findings that resource demands can shape attentional guidance by social cues. More critically, however, the nature of this shaping was different with head orientation cues than was the case with gaze cues in earlier experiments^25^. This suggests that conclusions drawn on the basis of results from studies using gaze cues are not necessarily broadly applicable to all social cues, and that different types of social cues differentially guide attention. It remains for future studies to more closely examine and confirm these disparities and establish the similarities and differences in underlying cognitive mechanisms.
